# Contralateral acupuncture versus ipsilateral acupuncture in the rehabilitation of post-stroke hemiplegic patients: a systematic review

**DOI:** 10.1186/1472-6882-10-41

**Published:** 2010-07-30

**Authors:** Mi-kyung Kim, Tae-Young Choi, Myeong Soo Lee, Hyangsook Lee, Chang-ho Han

**Affiliations:** 1 Department of Korean Internal Medicine, Dongguk University Ilsan Hospital, Goyang, South Korea; 2 Department of Oriental Medicine, Dongguk University Graduate School, Seoul, South Korea; 3 Division of Standard Research, Korea Institute of Oriental Medicine, Daejeon, South Korea; 4 Acupuncture and Meridian Science Research Center, College of Oriental Medicine, Kyung Hee University, Seoul, South Korea

## Abstract

**Background:**

Contralateral acupuncture (CAT) involves inserting needles in the meridian on the side opposite the disease location and is often used in post-stroke rehabilitation. The aim of this systematic review is to summarize and critically evaluate the evidence for and against the effectiveness of CAT for post-stroke rehabilitation as compared to ipsilateral acupuncture (IAT).

**Methods:**

Seventeen databases were searched from their inceptions through June 2010. Prospective clinical trials were included if CAT was tested as the sole treatment or as an adjunct to other treatments for post-stroke rehabilitation and compared to IAT.

**Results:**

Eight randomized clinical trials (RCTs) met our inclusion criteria. Four of them reported favorable effects of CAT compared to IAT for at least one outcome. A meta-analysis showed superior effects of CAT compared to IAT on recovery rate (n = 361; risk ratio (RR), 1.12; 95% confidence intervals (CIs), 1.04 to 1.22, P = 0.005). Subgroup analysis also showed favorable effects of using CAT on patients with cerebral infarction (n = 261; RR, 1.15; 95% CIs, 1.04 to 1.27, P = 0.006). Further analysis including patients with cerebral infarction and intracranial hemorrhage, however, failed to show these advantages (n = 100; RR, 1.11; 95% CIs, 0.85 to 1.46, P = 0.43).

**Conclusion:**

The results of our systematic review and meta-analysis suggest that there is limited evidence for CAT being superior to IAT in the treatment of cerebral infarction. The total number of RCTs included in our analysis was low, however, and the RCTs included had a high risk of bias. Future RCTs appear to be warranted.

## Background

Stroke is one of the most common causes of death in the world. Despite the considerable benefits of organized stroke care, many stroke survivors remain moderately or severely disabled [1,2]. In Western medicine, no single form of complementary or alternative medicine (CAM) is commonly used to manage post-stroke rehabilitation or recovery [3]. In East-Asian countries, however, acupuncture and herbal medicine are widely used [3]. A recent survey reported that 46% of stroke patients use some form of CAM such as herbal medicine, acupuncture-type treatments or chiropractic treatments [4]. Contralateral acupuncture (CAT), also called opposite needling or crossing needling, is a needling technique where acupuncture points on the right side are selected for diseases on the left and *vice versa*. The technique is commonly used and usually preferred to ipsilateral acupuncture (IAT) for treating post-stroke hemiplegia or sciatica [5]. Several studies have shown effects of CAT on pain [6,7], dizziness [8], herpes zoster [9], and stroke [10].

Although poor methodological quality and possibility of publication bias limits the strength of recommendation, the most recently published meta-analysis suggests that acupuncture may be an effective treatment for post-stroke rehabilitation [11]. In practice, CAT is often used as a treatment in post-stroke rehabilitation for hemiplegic patients in East-Asian Medicine hospitals and clinics. Thus, it seems pertinent to evaluate the effectiveness of CAT in post-stroke rehabilitation. To date, no systematic review has been performed on this topic. The aim of this systematic review, therefore, was to summarize and critically evaluate the evidence for or against the effectiveness of CAT for post-stroke rehabilitation of hemiplegic patients as compared to IAT.

## Methods

### Databases

The following databases were searched from their inceptions through June 2010: MEDLINE, EMBASE, CINAHL, AMED, Cochrane Central Register of Controlled Trials, Cochrane Database of Systematic Reviews, Database of Abstracts of Reviews of Effects, Cochrane Methodology Register, a Chinese medical database (CNKI), three Japanese medical databases (Journal Archive, Science Link Japan, and Japan Science & Technology link), 6 Korean medical databases (The Research Information Service System, Korean Studies Information, DBPIA, Korea Institute of Science and Technology Information, KoreaMed, and Korean National Assembly Library), and 4 major Korean traditional medical journals (Journal of Korean Oriental Internal Medicine, Journal of Oriental Rehabilitation Medicine, Journal of Sasang Constitutional Medicine and Journal of Korean Oriental Medicine). The following search terms were used: [("Contralateral acupuncture" OR "Contralateral needling" OR "Healthy side acupuncture" OR "Healthy side needling" OR "Opposite side acupuncture" OR "Opposite side needling") AND (stroke OR apoplexy OR cva OR cerebrovascular attack OR cerebrovascular accident OR cerebrovascular* OR cerebral infarction OR cerebral hemorrhage OR cerebral*). Furthermore, the references from all located articles were manually searched for additional relevant articles.

### Study selection

Prospective randomized clinical trials (RCTs) comparing the clinical effects of CAT to those of IAT were included. The studies involving post-stroke hemiplegic patients who were diagnosed clinically and/or by brain computed tomography (CT) scan or brain magnetic resonance imaging (MRI), including patients with cerebral infarction, intracerebral hemorrhage, cerebral embolism, or unclassified stroke, were included. To be included, acupuncture treatment on the affected side should be compared with that on the unaffected side. Trials where concomitant treatments were given were also included if they were given to both acupuncture and control groups. No language restrictions were imposed on study selection, and dissertations and abstracts were included provided they contained sufficient detail.

### Data extraction, quality control, and assessment of the risk of bias

All articles were read by two independent reviewers (MKK, TYC) who extracted data from each article according to predefined criteria. The risk of bias was assessed using the assessment tool for 'risk of bias' from the Cochrane Handbook for Systematic Reviews of Interventions [12]. The following characteristics were assessed: (1) Was the allocation sequence adequately generated? (2) Was allocation adequately concealed? (3) Was knowledge of the allocated interventions adequately prevented during the study? (4) Were incomplete outcome data adequately addressed? (5) Are reports of the study free of suggestion of selective outcome reporting? (6) Was the study free of other problems that could put it at a risk of bias?. Our review used 'Y, U, N' as keys of the judgments; the answer 'Yes' indicated a low risk of bias (Y), 'Unclear' indicated that a risk of bias is uncertain (U), and the answer 'No' indicated a high risk of bias (N).

Patient blinding was assumed when the control intervention was indistinguishable from acupuncture, even if the word "blinding" was not used in the report. Given that it is virtually impossible to blind therapists to the types of acupuncture, we assessed patient and assessor blinding separately. Disagreements were resolved by discussion between the two reviewers (MSL, HL). There were no disagreements between the two reviewers about the results.

### Data synthesis

To summarize the effects of acupuncture on patient outcomes as compared to baseline, we estimated weighted mean differences (WMD) or standardized mean differences (SMD) and 95% confidence intervals (CIs) for each study using the Cochrane Collaboration's software (Review Manager [RevMan] Version 5.0 for Windows. Copenhagen: The Nordic Cochrane Centre). Relative risk (RR) and 95% CIs were also calculated. The variance of change was determined using a correlation factor of 0.5. When appropriate (i.e., when excessive statistical heterogeneity did not exist), we pooled the data across studies using a random effects model. The chi-square, tau^2 ^and Higgins I^2 ^tests were used to assess heterogeneity.

## Results

### Study description

**Figure 1:**
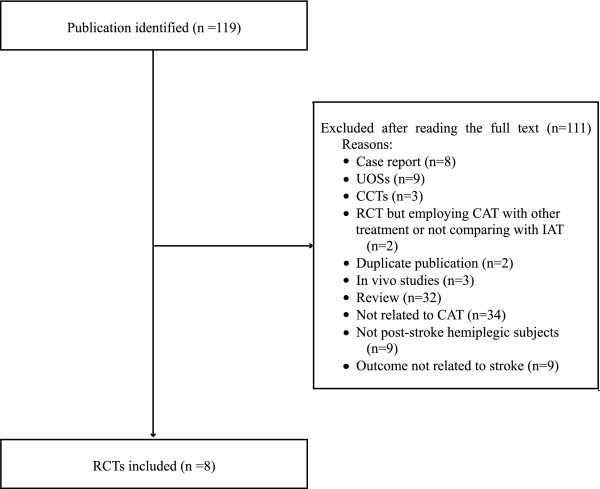
**Flow chart of the study selection process**. UOS: uncontrolled observational study; CCT: controlled clinical trial; RCT: randomized clinical trial; CAT: contralateral acupuncture; IAT: ipsilateral acupuncture.

The literature searches identified 119 potentially relevant articles, 8 of which were included in this review (Figure 1). Key data from the 8 included RCTs are summarized in Table 1[10,13-19]. Seven of the included trials were from China [13-19], and one was from Korea [10]. In addition, six of the included trials [10,13-17] followed a two-arm parallel group design, while two employed four-arm parallel group design [18,19]. The devices used for treatment were either electroacupuncture (EA) [14,15] or manual acupuncture [10,13,16-19]. In one trial, a big size needle, i.e. 0.4-1.6 mm × 150-500 mm, penetrating from one acupuncture point through another in CAT group was compared with IAT using filiform needles [15]. All of the included trials used CT scans and MRI to diagnose stroke. Four of the RCTs [13-16] included subjects with cerebral infarction, while the other four [10,17-19] included subjects with either cerebral infarction or intracranial hemorrhage.

**Table 1:** Key data from RCTs comparing contralateral acupuncture (CAT) to ipsilateral acupuncture (IAT) in post-stroke hemiplegic patients

First author (Year)	Sample size/Severity/Diagnosis	Groups	Main outcomes	Intergroup difference	CAT group	IAT group	Co-interventions for both groups	Risk of bias*
**Infarction**								
							
Pan (2009) [13]	53 n.r. CT scan or MRI	(A) CAT (n = 28) (B) IAT (n = 25)	1) Response rate 2) NDS	1) RR, 1.16 [0.93, 1.45], NS 2) MD, 3.69 [1.43, 5.95], P = 0.001	PC6, LI4, ST36, LR3 de-qi elicited, manipulation at every 5 min, 20 min per session, once daily for 10 days, interval of 5 days after one course, three courses in total	Identical points and procedures as CAT group	None	U-U-U-U-Y-Y-Y
Chen (2007) [14]	68 Mild to severe CT scan or MRI	(A) EA CAT (n = 34) (B) EA IAT (n = 34))	1) Response rate 2) NDS	1) RR, 1.07 [0.88, 1.31], NS 2) MD, 0.01 [-4.00, 4.02], NS	Points: LI15, LI11, LI10, TE5, LI4, ST31, GB31, GB34, ST36, ST41, GB36 1.7 Hz, for 30 min, once daily	Identical points and procedures as CAT group	Scalp acupuncture with manual twirling at 180-200 Hz, manipulation at every 10 min for 3 times, once daily for 30 days	U-U-U-U-U-Y-Y
Liu (2005) [15]	60 Mild (shoulder pain) CT scan or MRI	(A) Big size needle CAT (n = 30) (B) IAT (n = 30)	Response rate	RR, 1.17 [0.95, 1.43], NS	Points: GB34 through GB39 0.4-1.6 mm × 150-500 mm, de-qi elicited, manipulation at every 10 min, for 30 min, once daily, days n.r.	Points: LI15, SI9, LI14, LI11, TE5 0.30 mm × 40 mm, de-qi elicited, manipulation at every 10 min, for 30 min, once daily, days n.r.	Active and passive exercises	U-U-N-N-Y-Y-U
Sun (2000) [16]	80 n.r. CT scan or MRI	(A) CAT (n = 40) (B) IAT (n = 40)	Response rate	RR, 1.19 [1.00, 1.41], P = 0.05	Basic points: GV20, GB20, LI15, LI11, TE5, LI4, GB34, ST36, GB39, ST41 Additional points: GV26, PC8, LR3, BL18, BL23, BL17, SP10, ST40, SP6 De-qi elicited One course: 30 min, once daily for 10 sessions, interval of 2 days after one course Three courses in total	Identical points and procedures as CAT group	None	U-U-N-N-Y-Y-Y

**Infarction and hemorrhage mixed**								
						
Hong (2009) [17]	60 Severe (post-stroke shoulder-hand syndrome) CT scan or MRI	(A) CAT (n = 30) (B) IAT (n = 30)	1) Response rate 2) FMA 3) ADL 4) Pain (VAS)	1) RR, 1.04 [0.89, 1.21], NS 2) MD, 1.10 [0.06, 2.14], P = 0.04 3) MD, 10.67 [2.44, 18.90], P = 0.01 4) MD, -10.60 [-16.83, -4.37], P = 0.0009	1^st ^set of points: LI15, SI9, LI10, TE6, SI3, GB34, and most painful points 2^nd ^set of points: TE14, LI14, LI11, TE5, LI4, ST38, and most painful points 1^st ^and 2^nd ^sets in turn, 0.38 mm × 40-65 mm, de-qi elicited,2 Hz manipulation at every 10 min, One course: for 30 min, once daily for 10 sessions, interval of 2-3 days after one course Two courses in total	Identical points and procedures as CAT group	None	Y-U-N-N-Y-Y-Y
Ni (2009) [18]	80 n.r. CT scan or MRI	(A) CAT (n = 20) (B) IAT (n = 20) *(C) CAT +CSS (n = 20) *^†^ *(D) CSS (n = 20) *^†^	1) Response rate 2) FMA	1) RR, 1.33 [0.88, 2.03], NS 2) MD, 14.54 [9.42, 19.66], P < 0.0001	Points: PC6, LU5, LU4 for arms; ST36, GB34, LR3, GB40, GB31 for legs, taking turns every other day 20 min, once daily for 6 days, 4 weeks in total	Points: LI15, LI11, LI10, TE5, LI4 for difficult extension; LU5, PC3, PC6 for difficult flexion Identical procedures as CAT	None	Y-U-N-Y-Y-Y-Y
Huang (2008) [19]	120 Mild to severe CT scan or MRI	(A) CAT (n = 30) strong stimulation (B) CAT (n = 30) weak stimulation (C) IAT (n = 30) strong stimulation (D) IAT (n = 30) weak stimulation	1) FMA 2) ADL	1) A vs. C, MD, 2.50 [-2.43, 7.43], NS B vs. D, MD, -0.60[-5.78,4.58], NS 2) A vs. C, MD, 0.66[-7.14, 8.46], NS B vs. D, MD, -0.67[-5.46,4.12], NS	LI15, LI14, TE10, TE9, TE5, LI5, LI6, TE3 for arms; BL37, LR9, ST36, GB39, BL62, GB40, GB41 for legs 30 min, once daily for 6 days, one day rest, for 4 weeks Twisting and twirling to 180 and 90 degrees/lifting and thrusting to 5 and 3 mm for strong and weak stimulation, respectively	HT1, LU5, PC3, LI11, LI10, PC6, PC7, PC8 for arms; ST31, ST32, BL40, BL57, SP6, KI3, KI6, KI1 for legs Identical procedures as CAT group	None	Y-U-N-N-Y-Y-Y
Seo (2001) [10]	13 Moderate CT scan or MRI	(A) CAT (n = 7) (B) IAT (n = 6)	MBI	MD, -3.60 [-29.96, 22.76], NS	Points: GV20, CV24, GB20, LI11, LI4, TE5, LI10, ST36, GB34, GB31, GB39, LR3, Bafeng, Baxie on unaffected side; LI11, LI4, ST36, LR3 on affected side 20 min, once daily for 3 weeks	Points: GV20, CV24, GB20, LI11, LI4, TE5, LI10, ST36, GB34, GB31, GB39, LR3, Bafeng, Baxie on affected side; LI11, LI4, ST36, LR3 on unaffected side Rest of procedures identical as CAT group	None	U-U-N-Y-Y-Y-Y

ADL: Activity of Daily Living Scale; AT: Acupuncture therapy; CAT: Contralateral acupuncture (needling the unaffected side); CSS: continual static stretch; CT: Computed tomography; FMA: Fugl-Meyer Assessment; IAT: Ipsilateral acupuncture (needling the affected side); min: minute; MBI: modified Barthel Index; MD: mean difference; n.a.: not applicable; MRI: Magnetic resonance imaging; NDS: Neurological Deficit Score; n.r.: not reported; NS: not significant; RR: response rate; VAS: visual analogue scale. ^† ^We excluded this group because it was not comparable to the other groups.

*(1) Was the allocation sequence adequately generated? (2) Was allocation adequately concealed? (3) Was knowledge of the allocated interventions adequately prevented during the study (both to patient and outcome assessor)? (4) Were incomplete outcome data adequately addressed? (5) Are reports of the study free of suggestion of selective outcome reporting? (6) Was the study apparently free of other problems that could put it at a risk of bias?; (Y) indicates "Yes (low risk of bias)"; (U), "Unclear"; (N), "No (high risk of bias)".^12^

### Risk of Bias

Risk of bias assessment is shown in Table 1. Three of the included RCTs described the sequence generation [17-19] and they all used proper methods [10,17-19]. It was not clear whether group assignment was adequately concealed in any of the included trials. All of the included trials were rated as 'U' or 'N' for patient blinding. and two studies were rated as 'Y' for outcome assessor blinding [10,18]. For incomplete outcome data reporting, one study did not clearly reported how many patients were analyzed [14].

### Outcome Measures

#### Response rate

**Figure 2:**
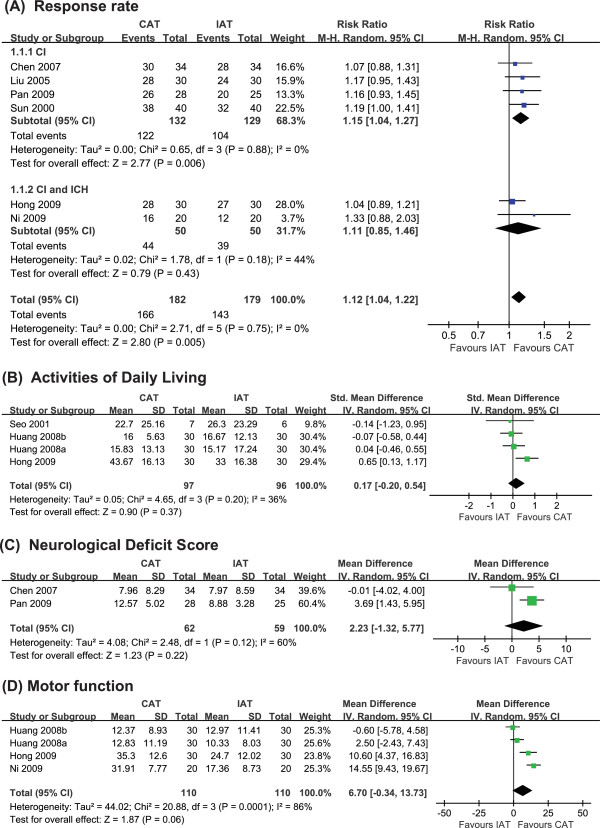
**Forest plot comparing contralateral acupuncture (CAT) to ipsilateral acupuncture (IAT) in terms of (A) response rate, (B) Activities of Daily Living, (C) Neurological Deficit Score, and (D) motor function in post-stroke hemiplegic patients**. CI: cerebral infaction; ICH: intracranial hemorrhage.

Six of the included trials examined the effects of CAT as compared to IAT on response rate [13-18]. One trial showed a favorable effect of CAT [16,18], while the others failed to do so. A meta-analysis, however, showed that CAT had superior effects compared to IAT on response rate (n = 361; RR, 1.12; 95% CIs, 1.04 to 1.22, P = 0.005; heterogeneity: χ^2 ^= 2.71, P = 0.75, I^2 ^= 0% Figure 2A). Subgroup analysis also showed favorable effects of CAT for patients with cerebral infarction (n = 261; RR, 1.15; 95% CIs, 1.04 to 1.27, P = 0.006; heterogeneity: χ^2 ^= 0.65, P = 0.88, I^2 ^= 0%) [13-16]. Further analysis including patients with cerebral infarction and intracranial hemorrhage, however, failed to find these favorable effects (n = 100; RR, 1.11; 95% CIs 0.85 to 1.46, P = 0.43; heterogeneity: χ^2 ^= 1.78, P = 0.18, I^2 ^= 44%) [17,18].

#### Activities of Daily Living (ADL)

Three of the RCTs assessed the effects of CAT on ADL compared to IAT [10,17,19]. One trial showed favorable effects of CAT [17], while the others did not [10,19]. A meta-analysis of these studies failed to show superior effects for CAT compared to IAT with regard to ADL (n = 193; SMD, 0.17; 95% CIs, -0.20 to 0.54, P = 0.37; heterogeneity: χ^2 ^= 4.65, P = 0.20, I^2 ^= 36% Figure 2B).

#### Neurological Deficit Score (NDS)

Two of the RCTs assessed the effects of EA-CAT compared to EA-IAT on NDS [13,14]. One trial showed the significant superior effects of EA-CAT [13], while the other one failed to do so[14]. A meta-analysis failed to show significant difference between the two methods (n = 121; WMD, 2.23; 95% CIs, -1.35 to 5.77, P = 0.22; heterogeneity: χ^2 ^= 2.48, P = 0.12, I^2 ^= 60% Figure 2C).

#### Motor function

Three RCTs tested the effects of CAT for motor function on Fugl-Meyer Assessment (FMA) [17-19]. Two trials showed favorable effects of CAT [17,18], while the other did not [19]. A meta-analysis of these studies failed to show superior effects for CAT compared to IAT (n = 220; WMD, 6.70; 95% CIs, -0.34 to 13.73, P = 0.06) with heterogeneity (χ^2 ^= 20.88, P = 0.0.001, I^2 ^= 86% Figure 2D). 

## Discussion

Few rigorous RCTs testing the effects of CAT for post-stroke rehabilitation are currently available, and the existing studies do not provide much information regarding the superiority of CAT over IAT for patients with post-stroke hemiplegia. Our meta-analysis of 6 trials demonstrated that CAT may be superior to IAT in post-stroke rehabilitation [13-18]. The total number of RCTs and total sample size included in our analysis, however, were too small to draw firm conclusions about the superiority of CAT.

Only four [13,16-18] of the 8 RCTs evaluated here reported favorable effects of CAT compared to IAT for at least one outcome measure. A meta-analysis showed that CAT had superior effects compared to IAT on response rate in patients with cerebral infarction [13-16] but failed to do so in a group of mixed patients with cerebral infarction or cerebral infarction [17,18]. Moreover, clinical trials should follow the CONSORT guidelines.

There are many prognostic factors for post-stroke rehabilitation, including type of stroke, size of the brain lesion, interval to onset, severity of symptoms, age, gender, and past history. Few of the trials included here, however, described these factors, and none of them conducted subgroup analysis for each prognostic factor. The stroke stage (acute or chronic) was also not reported. Outcomes were measured using ambiguous scales for many of the trials, despite the fact that it is important to select published or validated measurement scales. None of the trials conducted a follow-up assessment after treatment or reported adverse events or patient acceptance of acupuncture.

In the view of East-Asian Medicine, the Yellow Emperor Neijing states that, "if someone has disease related with the left side, the treatment point is the right side, and vice versa," emphasizing the importance of treatment side [5,20]. The clinical implications of this study may involve the selection of acupuncture points for treating stroke patients, which is thought to be one of the key issues in therapeutic effectiveness of acupuncture. In this review, there was a significant difference between CAT and IAT in terms of response rate; however, high risk of bias in the included trials prevents us from making firm conclusions. Not a single trial in our review adequately concealed group allocation and 5 out of 8 trials did not clearly reported how randomization was conducted. This raises concerns about validity of the results from these trials hence limits their applicability. In comparison of CAT with IAT in post-stroke hemiplegia, it is almost impossible to blind patients as demonstrated in our risk of bias assessment. Future trials then should pay more attention to adopting blinded outcome evaluation using validated scales rather than arbitrarily categorized response rate. One interesting finding of our review is that there was no between group difference in ADL or motor function on FMA while response rate in CAT was significantly better than IAT. It is not difficult to attribute this finding to failed blinding of included trials.

Assuming that CAT is beneficial for post-stroke rehabilitation, its mechanism of action may be of interest. The postulated modes of action involve changes in brain activity according to treatment side [20] and direct effects on target organs and the central nervous system [21-23]. None of these theories, however, has been established in the literature.

This systematic review has several limitations. First, relevant RCTs may have been overlooked despite our best efforts to conduct sensitive literature searches without language restrictions. Second, all included RCTs were conducted in China and Korea, and studies from these countries are known to exhibit a very low rate of negative results [24]. In fact, the distorting effects of publication bias and location bias have been well-documented [25,26]. Finally, the reviewed studies had a high risk of bias; therefore, the results of this review might be exaggerated. Methodological shortcomings such as inadequate allocation concealment, small sample size and inadequate blinding require further large studies that incorporate methodological rigor.

## Conclusion

The results of our systematic review and meta-analysis suggest that there is limited evidence of CAT being superior to IAT in post-stroke rehabilitation. The paucity of RCTs included in our analysis, coupled with small sample size and high risk of bias, prevents us from drawing firm conclusions about the effectiveness of CAT. Future rigorous studies are required to confirm our limited findings.

## Conflicts of interests

The authors declare that they have no competing interests.

## Authors' contributions

MKK and CHH conceived the study design. MKK, TYC and MSL searched and selected the trials and extracted, analyzed and interpreted the data. MKK and MSL drafted the manuscript. TYC and HSL updated the search and the content of the review. MSL, HSL and CHH helped with the study design and critically reviewed the manuscript. All authors read and approved the final version of the manuscript.

## Pre-publication history

The pre-publication history for this paper can be accessed here:

http://www.biomedcentral.com/1472-6882/10/41/prepub
